# Effect of Soret diffusion on the growth of spherical crystals in supercooled alloy melts under oscillatory flow

**DOI:** 10.1371/journal.pone.0313150

**Published:** 2024-11-04

**Authors:** Xiaoxia Liu, Hailong Fan, Yanyan Shan

**Affiliations:** College of science, Inner Mongolia University of Technology, Hohhot, China; University of Salento, ITALY

## Abstract

The growth of spherical crystals in binary alloy melts with thermal diffusion effects under oscillatory flow is investigated analytically. Using the multiple scale method, we derive approximate analytical solutions for both the crystal interface growth rate and the solute concentration. Our results demonstrate that the Soret effect significantly influences both the solute concentration near the crystal interface and the crystal growth rate. Specifically, with a positive Soret coefficient, the growth rate of spherical crystals in a binary dilute alloy melt decreases as the coefficient increases, while the solute concentration near the interface increases. In contrast, with a negative Soret coefficient, the growth rate of the spherical crystals increases as the coefficient decreases, and the solute concentration near the interface decreases. Additionally, the presence of oscillatory flow markedly promotes the grain refinement induced by the Soret effect.

## Introduction

Crystal growth technology not only explains natural phenomena like snowflakes but is also widely applied in fields such as semiconductors, optics, medicine and new materials. The development of strong and tough dual-enhanced metal materials is crucial for manufacturing in high speed and heavy haul railways, national defense and intelligent equipment. Controlling the microstructure of crystals and their growth dynamics is a key technical approach to solving the inverse relationship between strength and toughness in metal materials. During melt solidification, the homogenization of composition and structure and grain refinement are forefront issues in developing these materials. Scholars have studied factors like anisotropic surface tension [1–3], interface dynamics [4–7], flow [8–10], and magnetic fields [11] on the growth of spherical grains. The shape of crystals is mainly determined by heat and mass transfer in their growth environment, beyond their internal lattice structures. According on Fick’s law, the diffusion flux of the solute in the binary alloy is proportional to the concentration gradient of the solute. In addition to concentration gradient, coupled with the temperature gradient, the modified Fick’s law ***J*** = −*D*_*L*_∇*C*_*L*_ − *D*_*T*_(*β*_0_ + *β*_1_*C*_*L*_)∇*T*_*L*_ [12] is obtained, where *D*_*L*_ and *D*_*T*_ are the isothermal diffusion and thermal diffusion coefficients of the solute, respectively. The constants *β*_0_ and *β*_1_ represent binary values (0 or 1). The ratio *D*_*T*_/*D*_*L*_ is the Soret coefficient whose value is of the order of 10^−2^K^−1^ to 10^−3^K^−1^ and its value can be either positive or negative [13]. The Soret effect, induced by temperature gradient, is also referred to as thermomigration in the solidification processes of metals and semiconductors. Even at small temperature gradients, since the diffusion flux determines the solute gradient in the melt, the Soret effect significantly impacts both the morphological stability of solid-liquid interface and the stability of fluid dynamics. Additionally, the Soret effect alters the governing equations of crystal growth, it is essential to study its influence on the crystal growth process. For example, Hurle [14] examined the growth of crystals in binary dilute alloy melts, concluding that the Soret coefficient does not significantly alter the interface morphology of the crystal. Vaerenbergh and Coriell et al. [15] investigated effect of the Soret effect on the morphological stability of crystals, and demonstrated that the sign of the Soret coefficient determines morphological stability. Rahman et al. [16] provided a comprehensive review of experimental and theoretical studies on the Soret effect. Jafar-Salehi et al. [17] studied the effect of thermal diffusion on the solidification of molten metal alloys, and discovered that thermodiffusion accelerates solidification rates and affects liquid composition and microstructure formation, depending on the Soret coefficient’s magnitude. Sassi et al. [18] studied the effect of Soret effect on solute segregation during the growth process of semiconductor materials. The results showed that the Soret effect significantly influences composition uniformity of crystal in semiconductor growth, with the Soret coefficient’s value and sign playing important roles. Chen et al. [19] further explored effect of Soret effect on the growth of spherical crystals, finding that a positive Soret coefficient accelerates the rate of crystal growth, while a negative Soret coefficient decelerates it. Yang et al. [20] studied the effect of the Soret effect on the melting of nanoparticles in alloy melts, discovering that a positive Soret coefficient increases solute concentration near the interface, whereas a negative Soret coefficient reduces it.

Since flow can significantly alter heat and mass transfer processes in the liquid phase, researchers have explored the Soret effect on flow. Zimmermann et al. [21] studied Benard convection in binary mixtures with solidification and the Soret effect, finding that a negative Soret coefficient stabilizes convection. Hayat et al. [22] examined the influence of the Soret effect on radiative three-dimensional flow, showing that smaller Soret coefficients result in lower concentrations near the boundary layer. Mishra et al. [23] investigated the Soret effect on unsteady magnetohydrodynamics(MHD) mixed convection in porous media, revealing that fluid velocity in the boundary layer increases with a higher Soret number. Gangadharaiah [24] analyzed surface-tension-driven convection in a fluid layer overlying an anisotropic porous layer, incorporating the Soret effect. He found that the Soret effect stabilizes flow in anisotropic porous media, with higher Soret coefficients expanding the stable region. Raju et al. [25] explored the effect of the Soret effect on unsteady MHD convective flow of Jeffrey nanofluid passing through porous boundary layers. They observed that both flow velocity and concentration near the boundary layer increased with a higher Soret coefficient. Lin et al. [26] studied the effect of the Soret effect on heat and mass transfer in Marangoni flow within the boundary layer of a rotating disk, finding that the Soret coefficient slows the decrease of fluid concentration in the boundary layer. These studies indicate that the Soret effect influences fluid stability, flow velocity and solute concentration near fixed boundary layers. Inspired by these findings, it is necessary to investigate influence of the Soret effect on the growth rate and morphology of crystals when there is flow in the melt.

In practice, electromagnetic or vibrational forces often induce oscillatory flow in the liquid phase to promote solute homogenization and grain refinement. For example, Schulze et al. [27] analyzed interface stability during directional solidification of dilute binary alloys, showing that stability is affected by oscillation frequency and amplitude. Parambil et al. [28] found that nucleation rates increase with flow speed in oscillatory flow. Chen et al. [29] used asymptotic analysis to show that oscillatory flow alternately promotes and inhibits grain growth, leading to refinement. Thus, oscillatory flow is a significant method for refining and homogenizing grains, and the Soret effect remains relevant in this process.

This study focuses on the Soret effect on the growth of spherical grains in undercooled alloy melts subjected to oscillatory flow. Previous research by Chen et al. [19] investigated the Soret effect on crystal growth in the absence of flow, while Raju et al. [25] examined impact of the Soret effect on flow velocity and concentration near boundaries, but did not address grain growth. Building on these researches, we analyze combined effects of the Soret effect and oscillatory flow on spherical grain growth. The multiple scale method is employed to derive approximate expressions for interface growth speed and solute concentration.

## Mathematical model

The growth of a single spherical crystal in an undercooled binary alloy melt with the Soret effect under oscillatory flow is considered. The crystal-melt interface of the particle is expressed as *R* = *R*(*θ*, *φ*, *t*) in the spherical coordinate frame (*r*, *θ*, *φ*) whose origin is set at the center of the sphere, where *r* is the radial distance, *θ* is the elevation angle and *φ* is the azimuthal angle. *T*_*L*_ and *T*_*S*_ are used to represent temperatures of the liquid phase and the solid phase. The concentrations in the liquid phase and the solid phase are expressed as *C*_*L*_ and *C*_*S*_ respectively, *T*_∞_ and *C*_∞_ represent the far-field temperature and far-field concentration of the liquid phase. The meanings of symbols such as Δ*T* and Δ*H* et al. can be found in the S1 Appendix. The solute diffusion equation with the Soret effect in the liquid phase is expressed as ***U***_*L*_ ⋅ ∇*C*_*L*_ + ∂*C*_*L*_/∂*t* = −∇ ⋅ ***J***, where the flow field velocity ***U***_*L*_ is caused by the oscillatory flow ***U***_*L*_ = −*A*_*D*_ cos Ω*t* ***k*** far away from the spherical crystal, *A*_*D*_ represents the amplitude, Ω denotes the frequency, and ***k*** is the third unit vector of the Cartesian rectangular coordinates. The initial radius *r*_0_ is selected as the length scale, the characteristic velocity *V* = *k*_*L*_Δ*T*/(*r*_0_Δ*H*) is the velocity scale, *r*_0_/*V* is the time scale, Δ*H*/*c*_*p*_*ρ*_*L*_ is the temperature scale, *C*_*e*_ − *C*_*S*_ is the concentration scale, Δ*T* is the undercooling defined as Δ*T* = *T*_*e*_−*T*_∞_, *T*_*e*_ is the liquid phase equilibrium temperature and *C*_*e*_ is the liquid phase equilibrium concentration of the flat interface, Δ*H* is the latent heat per unit volume, *c*_*p*_ is the specific heat, and *ρ*_*L*_ is the melt density. The following dimensionless quantities are introduced

$$\begin{array}{c} {\bar{C}}_{L}=\frac{C_{L}-C_{e}}{C_{e}-C_{s}},\;{\bar{T}}_{L}=\frac{T_{L}-T_{e}}{\Delta H/(c_{p}\rho_{L})},\;{\bar{T}}_{S}=\frac{T_{S}-T_{e}}{\Delta H/(c_{p}\rho_{L})},\;\bar{r}=\frac{r}{r_{0}},\;\bar{t}=\frac{t}{r_{0}/V}, \end{array}$$

$$\begin{array}{c} {\bar{U}}_{L}=\frac{U_{L}}{V}=\frac{1}{V}\left(u,v,w\right),\;V=\frac{k_{L}\Delta T}{r_{0}\Delta H},\;\bar{P}=\frac{P}{k_{L}V/r_{0}c_{p}},\;{\bar{U}}_{I}=\frac{U_{I}}{V}. \end{array}$$

For convenience, omit the ‘ - ’ on the dimensionless quantity. The dimensionless equations governing the crystal growth system are thermal diffusion, solute diffusion, Navier-Stokes and continuity equations

$$\begin{array}{c} \varepsilon \left(\frac{\partial T_{L}}{\partial t}+U_{L}\cdot \nabla T_{L}\right)=\nabla^{2}T_{L}, \end{array}$$ (1)

$$\begin{array}{c} \varepsilon \lambda_{t}\frac{\partial T_{S}}{\partial t}=\nabla^{2}T_{S}, \end{array}$$ (2)

$$\begin{array}{cc} \varepsilon \lambda_{c}\left(U_{L}\cdot \nabla C_{L}+\frac{\partial C_{L}}{\partial t}\right) & =\nabla^{2}C_{L}+\lambda_{c}S_{T}^{\left(1\right)}\nabla^{2}T_{L}+S_{T}^{\left(2\right)}\nabla C_{L}\nabla T_{L}+S_{T}^{\left(2\right)}C_{L}\nabla^{2}T_{L}\\ & +\lambda_{c}S_{T}^{\left(3\right)}\nabla^{2}T_{L}, \end{array}$$ (3)

$$\begin{array}{c} \varepsilon \left(U_{L}\cdot \nabla \right)U_{L}=-\nabla P+\text{Pr}\nabla^{2}U_{L}, \end{array}$$ (4)

$$\begin{array}{c} \nabla \cdot U_{L}=0, \end{array}$$ (5)

where *ε* is the dimensionless relative undercooling parameter, λ_*c*_ and λ_*t*_ are respectively the ratio of thermal diffusivity to solute diffusivity in the liquid and solid phase, $S_{T}^{\left(1\right)}$, $S_{T}^{\left(2\right)}$ and $S_{T}^{\left(3\right)}$ are three modified Soret coefficients,

$$\begin{array}{c} \varepsilon =\frac{\Delta T}{\Delta H/(c_{p}\rho_{L})},\;\lambda_{t}=\frac{\kappa_{L}}{\kappa_{S}},\;\lambda_{c}=\frac{\kappa_{L}}{D_{L}},\;S_{T}^{\left(1\right)}=\frac{\beta_{0}\Delta HD_{T}}{c_{p}\rho_{L}\kappa_{L}\left(C_{e}-C_{S}\right)}, \end{array}$$

$$\begin{array}{c} S_{T}^{\left(2\right)}=\frac{\beta_{1}\Delta HD_{T}}{c_{p}\rho_{L}D_{L}},\;S_{T}^{\left(3\right)}=\frac{D_{T}\beta_{1}C_{e}\Delta H}{\left(C_{e}-C_{S}\right)c_{p}\rho_{L}\kappa_{L}},\;\text{Pr}=\frac{\upsilon }{\kappa_{L}}. \end{array}$$

At the interface *R* = *R*(*θ*, *φ*, *t*), it meets with the dimensionless mass conservation condition, the segregation condition, the energy conservation condition, the thermal equilibrium condition and Gibbs-Thomson condition

$$\begin{array}{c} \varepsilon \lambda_{c}S_{k}U_{I}=-\nabla C_{L}\cdot n-\lambda_{c}S_{T}^{\left(1\right)}\nabla T_{L}\cdot n-S_{T}^{\left(2\right)}C_{L}\nabla T_{L}\cdot n-\lambda_{c}S_{T}^{\left(3\right)}\nabla T_{L}\cdot n, \end{array}$$ (6)

$$\begin{array}{c} C_{L}=-\frac{1}{k}-\frac{k-1}{k}C_{es}, \end{array}$$ (7)

$$\begin{array}{c} \varepsilon U_{I}=\left(k_{t}\nabla T_{S}-\nabla T_{L}\right)\cdot n, \end{array}$$ (8)

$$\begin{array}{c} T_{S}=T_{L}, \end{array}$$ (9)

$$\begin{array}{c} T_{I}=2\varepsilon \Gamma k-M_{c}\lambda_{d}C_{L}-M_{e}\varepsilon -\varepsilon E^{-1}M_{k}U_{I}, \end{array}$$ (10)

where

$$\begin{array}{c} S_{K}=\frac{\left(1-k\right)C_{S}}{k(C_{e}-C_{S})},\;C_{es}=\frac{C_{e}}{C_{e}-C_{S}},\;k_{t}=\frac{k_{s}}{k_{L}},\;\lambda_{d}=\frac{D_{L}}{\kappa_{L}},\;E=\frac{\Delta T}{T_{M}}, \end{array}$$

$$\begin{array}{c} M_{K}=\frac{V}{\mu T_{M}},\;M_{C}=-\frac{m(C_{e}-C_{S})}{\Delta H/(c_{p}\rho_{L})},\;M_{e}=-\frac{m(C_{e}-C_{\infty })}{\Delta T},\;\Gamma =\frac{\gamma_{0}T_{M}}{r_{0}\Delta H\Delta T}, \end{array}$$

***n*** is the unit normal vector to the interface, *γ*_0_ is the isotropic surface energy.

The temperature field, concentration field, and fluid velocity also need to meet the following far-field conditions, as *r* → ∞

$$\begin{array}{c} C_{L}\to -C_{L,\infty }\lambda_{c}\varepsilon ,\;T_{L}\to -\varepsilon ,\;U_{L}\to -A_{m}\;\text{cos}\;\omega tk, \end{array}$$ (11)

where *A*_*m*_ = *A*_*D*_/*V* and *ω* = *a*Ω/*V* are the amplitude and frequency of the oscillatory flow, respectively.

The initial conditions are expressed as following, at *t* = 0,

$$\begin{array}{c} C_{L}{|}_{t=0}=-C_{L,\infty }\lambda_{c}\varepsilon ,\;T_{L}{|}_{t=0}=-\varepsilon ,\;T_{S}{|}_{t=0}=\varepsilon T_{S}^{*},\;R\left(\theta ,\varphi ,0\right)=1, \end{array}$$ (12)

where

$$\begin{array}{c} C_{L,\infty }=\frac{D_{L}\left(C_{e}-C_{\infty }\right)\Delta H}{\kappa_{L}c_{p}\rho_{L}\left(C_{e}-C_{S}\right)\Delta T}, \end{array}$$

and $T_{S}^{*}$ is the temperature of the crystal in the solid phase at *t* = 0.

## Asymptotic solution

To determine the asymptotic solution for the crystal growth model described by Eqs (1)–(12), we first need to analyze the orders of magnitude of the primary physical quantities involved. This analysis helps in determining the form of the asymptotic solution. For typical metals Δ*H*/(*c*_*p*_*ρ*_*L*_) is generally on the order of several hundred K. For instance, in a Cu-Fe alloy, Δ*H* = 2.4 × 10^9^Jm^−3^, *c*_*p*_ = 843 J kg^−1^K^−1^, *ρ*_*L*_ = 7940 kg m^−3^, Δ*H*/(*c*_*p*_*ρ*_*L*_) = 358.6 K. Since undercooling in the melt is typically on the order of several tens of degrees, the relative undercooling parameter *ε* is practically small, which is consistent with viewpoint of Xu [30]. The parameters *C*_*L*,∞_ and Γ are of the order of unity, while *M*_*c*_, *M*_*e*_, $S_{T}^{\left(1\right)}$, $S_{T}^{\left(2\right)}$ and $S_{T}^{\left(3\right)}$ are no more than unit order of magnitude.

According to the solidification theory, heat transport and solute transport are quite different near the interface and away from the interface, that is, there are two spatial scales. Therefore, the slow variable *ρ* = *εr* is introduced, and *r*, *ρ*, *θ*, *φ*, *t* are regarded as independent variables. Obviously, there is the following relationship ∂/∂*r* → ∂/∂*r* + *ε*∂/∂*ρ*, we use the multi-scale method to find the asymptotic solution of the crystal growth system given by Eqs (1)–(12).

In the region near the interface, expand as follows

$$\begin{array}{cccccc} C_{L} & =\varepsilon \lambda_{c}C_{L0}+\varepsilon^{2}\lambda_{c}C_{L1}+\cdots ,\; & U_{L} & =U_{L0}+\varepsilon U_{L1}+\cdots ,\; & T_{L} & =\varepsilon T_{L0}+\varepsilon^{2}T_{L1}+\cdots ,\\ T_{S} & =\varepsilon T_{S0}+\varepsilon^{2}T_{S1}+\cdots ,\; & P & =P_{L0}+\varepsilon P_{L1}+\cdots ,\; & Q & =Q_{0}+\varepsilon Q_{1}+\cdots ,\\ R & =R_{0}+\varepsilon R_{1}+\cdots , \end{array}$$ (13)

where *Q* = *εR*, *Q*_0_ = *εR*_0_, *Q*_1_ = *εR*_1_.

Substituting Eq (13) into Eqs (1)–(12) of crystal growth system and comparing the order of *ε*^*n*^, we can get the approximate equation of each order.

The equations for the leading order approximations are as following

$$\begin{array}{c} \nabla^{2}C_{L0}+S_{T}^{\left(1\right)}\nabla^{2}T_{L0}+S_{T}^{\left(3\right)}\nabla^{2}T_{L0}=0, \end{array}$$ (14)

$$\begin{array}{c} \nabla^{2}T_{L0}=0, \end{array}$$ (15)

$$\begin{array}{c} \nabla^{2}T_{S0}=0, \end{array}$$ (16)

$$\begin{array}{c} \nabla \cdot U_{L0}=0, \end{array}$$ (17)

$$\begin{array}{c} \text{Pr}\;\nabla^{2}U_{L0}-\nabla P_{L0}=0. \end{array}$$ (18)

The crystal growth system is subjected to the following interface conditions, at the interface *R* = *R*_0_(*t*),

$$\begin{array}{c} S_{k}\frac{dR_{0}}{dt}=-\frac{\partial C_{L0}}{\partial r}-S_{T}^{\left(1\right)}\frac{\partial T_{L0}}{\partial r}-S_{T}^{\left(3\right)}\frac{\partial T_{L0}}{\partial r}, \end{array}$$ (19)

$$\begin{array}{c} C_{L0}=-\frac{1}{k}-\frac{k-1}{k}C_{es}, \end{array}$$ (20)

$$\begin{array}{c} \frac{dR_{0}}{dt}=k_{t}\frac{\partial T_{S0}}{\partial r}-\frac{\partial T_{L0}}{\partial r}, \end{array}$$ (21)

$$\begin{array}{c} T_{S0}=T_{L0}, \end{array}$$ (22)

$$\begin{array}{c} T_{S0}=-\frac{2\Gamma }{R_{0}}-M_{c}C_{L0}-M_{e}-E^{-1}M_{k}\frac{dR_{0}}{dt}. \end{array}$$ (23)

The far-field conditions follow that, as *r* → ∞,

$$\begin{array}{c} C_{L0}\to -C_{L,\infty },\;T_{L0}\to -1,\;U_{L0}\to \left(-A_{m}\;\text{cos}\;\omega t\;\text{cos}\;\theta ,A_{m}\;\text{cos}\;\omega t\;\text{sin}\;\theta ,0\right). \end{array}$$ (24)

The initial conditions follow that, at *t* = 0,

$$\begin{array}{c} R_{0}\left(0\right)=1. \end{array}$$ (25)

The leading order solutions of temperature field, concentration field and flow field are expressed as

$$\begin{array}{c} T_{L0}=-1+\frac{dR_{0}}{dt}R_{0}^{2}\frac{1}{r}\left(e^{Q_{0}-\rho }\right), \end{array}$$ (26)

$$\begin{array}{c} T_{S0}=-1+\frac{dR_{0}}{dt}R_{0}, \end{array}$$ (27)

$$\begin{array}{c} C_{L0}=-C_{L,\infty }+\left(S_{k}-S_{T}^{\left(1\right)}-S_{T}^{\left(3\right)}\right)\frac{dR_{0}}{dt}R_{0}^{2}\frac{1}{r}\left(e^{Q_{0}-\rho }\right), \end{array}$$ (28)

$$\begin{array}{c} u_{0}=A_{m}\left(-\frac{R_{0}^{3}}{2r^{3}}-\frac{3R_{0}}{2r}+1\right)\;\text{cos}\;\omega t\;\text{sin}\;\theta , \end{array}$$ (29)

$$\begin{array}{c} v_{0}=A_{m}\left(-\frac{R_{0}^{3}}{4r^{3}}-\frac{3R_{0}}{4r}+1\right)\;\text{cos}\;\omega t\;\text{sin}\;\theta , \end{array}$$ (30)

$$\begin{array}{c} P_{L0}=\frac{3A_{m}\;\text{Pr}\;R_{0}}{2r^{2}}\;\text{cos}\;\omega t\;\text{cos}\;\theta , \end{array}$$ (31)

where ***U***_*L*0_ = (*u*_0_, *v*_0_, 0).

According to the interface conditions, crystal radius *R*_0_ satisfies the following ordinary differential equation

$$\begin{array}{c} \frac{dR_{0}}{dt}=\frac{R_{0}-2\Gamma }{R_{0}\left[R_{0}+E^{-1}M_{k}+M_{c}R_{0}\left(S_{k}-S_{T}^{\left(1\right)}-S_{T}^{\left(3\right)}\right)\right]}. \end{array}$$ (32)

Hence, the implicit solution of Eq (32) yields

$$\begin{array}{cc} t & =2\Gamma \left[2\Gamma +E^{-1}M_{k}+2\Gamma M_{c}\left(S_{k}-S_{T}^{\left(1\right)}-S_{T}^{\left(3\right)}\right)\right]\;\text{ln}\;\frac{R_{0}-2\Gamma }{1-2\Gamma }+[2\Gamma +E^{-1}M_{k}\\ & +2\Gamma M_{c}\left(S_{k}-S_{T}^{\left(1\right)}-S_{T}^{\left(3\right)}\right)]\left(R_{0}-1\right)+\frac{1}{2}\left[1+M_{C}\left(S_{K}-S_{T}^{\left(1\right)}-S_{T}^{\left(3\right)}\right)\right]\left(R_{0}^{2}-1\right). \end{array}$$ (33)

It can be seen from Eq (32) that the crystal grows at *dR*_0_/*dt* > 0, and the crystal shrinks at *dR*_0_/*dt* < 0.

The equations for the first order approximations take the following forms

$$\begin{array}{c} \frac{\partial T_{L0}}{\partial t}+U_{L0}\cdot \nabla T_{L0}=\nabla^{2}T_{L1}+2\frac{\partial^{2}T_{L0}}{\partial r\partial \rho }+\frac{2}{r}\frac{\partial T_{L0}}{\partial \rho }, \end{array}$$ (34)

$$\begin{array}{c} \lambda_{t}\frac{\partial T_{S0}}{\partial t}=\nabla^{2}T_{S1}+2\frac{\partial^{2}T_{S0}}{\partial r\partial \rho }+\frac{2}{r}\frac{\partial T_{S0}}{\partial \rho }, \end{array}$$ (35)

$$\begin{array}{c} \lambda_{c}(U_{L0}\cdot \nabla C_{L0}+\frac{\partial C_{L0}}{\partial t})=\nabla^{2}C_{L1}+2\frac{\partial^{2}C_{L0}}{\partial r\partial \rho }+\frac{2}{r}\frac{\partial C_{L0}}{\partial \rho }+\left(S_{T}^{\left(1\right)}+S_{T}^{\left(3\right)}\right)(\nabla^{2}T_{L1}\\ +2\frac{\partial^{2}T_{L0}}{\partial r\partial \rho }+\frac{2}{r}\frac{\partial T_{L0}}{\partial \rho })+S_{T}^{\left(2\right)}\nabla C_{L0}\nabla T_{L0}+S_{T}^{\left(2\right)}C_{L0}\nabla^{2}T_{L0}. \end{array}$$ (36)

The crystal growth system satisfies the following interface conditions

$$\begin{array}{cc} S_{K}\frac{\partial R_{1}}{\partial t}= & -\frac{\partial C_{L1}}{\partial r}+\left(S_{K}-S_{T}^{\left(1\right)}-S_{T}^{\left(3\right)}\right)\left[\frac{dR_{0}}{dt}R_{0}-\frac{2}{R_{0}}\frac{dR_{0}}{dt}R_{1}-Q_{1}\frac{dR_{0}}{dt}\right]\\ & -\left(S_{T}^{\left(1\right)}+S_{T}^{\left(3\right)}\right)\frac{\partial T_{L1}}{\partial r}-\left(S_{T}^{\left(1\right)}+S_{T}^{\left(3\right)}\right)\left[-R_{0}\frac{dR_{0}}{dt}+R_{1}\frac{2}{R_{0}}\frac{dR_{0}}{dt}+Q_{1}\frac{dR_{0}}{dt}\right]\\ & -S_{T}^{\left(2\right)}\left[\left(-C_{L,\infty }+\left(S_{K}-S_{T}^{\left(1\right)}-S_{T}^{\left(3\right)}\right)R_{0}\frac{dR_{0}}{dt}\right)\left(-\frac{dR_{0}}{dt}\right)\right], \end{array}$$ (37)

$$\begin{array}{c} \frac{\partial R_{1}}{\partial t}=k_{t}\frac{\partial T_{S1}}{\partial r}-\frac{\partial T_{L1}}{\partial r}-\frac{2}{R_{0}}\frac{dR_{0}}{dt}R_{1}+R_{0}\frac{dR_{0}}{dt}-\frac{dR_{0}}{dt}Q_{1}, \end{array}$$ (38)

$$\begin{array}{c} T_{L1}=T_{S1}+\frac{dR_{0}}{dt}R_{1}+R_{0}\frac{dR_{0}}{dt}Q_{1}, \end{array}$$ (39)

$$\begin{array}{cc} T_{S1}= & \frac{\Gamma }{R_{0}^{2}}\left(\Lambda +2\right)R_{1}-M_{c}C_{L1}-E^{-1}M_{k}\frac{\partial R_{1}}{\partial t}+\left(S_{k}-S_{T}^{\left(1\right)}-S_{T}^{\left(3\right)}\right)\\ & \cdot M_{c}\left(\frac{dR_{0}}{dt}R_{1}+R_{0}\frac{dR_{0}}{dt}Q_{1}\right), \end{array}$$ (40)

where

$$\begin{array}{c} \Lambda =\frac{1}{\text{sin}\;\theta }\frac{\partial }{\partial \theta }\left(\text{sin}\;\theta \frac{\partial }{\partial \theta }\right)+\frac{1}{{\text{sin}}^{2}\;\theta }\frac{\partial^{2}}{\partial \varphi^{2}}. \end{array}$$

The far field conditions are as follows, as *r* → ∞,

$$\begin{array}{c} \;C_{L1}\to 0,\;T_{L1}\to 0. \end{array}$$ (41)

The initial conditions are as follows, at *t* = 0,

$$\begin{array}{c} \;R_{1}\left(\theta ,\varphi ,0\right)=0. \end{array}$$ (42)

The first-order approximate solutions of temperature field, concentration field and crystal radius can be obtained as follows

$$\begin{array}{c} T_{L1}=T_{L1}^{*}+\frac{A_{0,0}}{r}+\frac{A_{1,0}}{r^{2}}\;\text{cos}\;\theta , \end{array}$$ (43)

$$\begin{array}{c} T_{S1}=T_{S1}^{*}+B_{0,0}+rB_{1,0}\;\text{cos}\;\theta , \end{array}$$ (44)

$$\begin{array}{c} C_{L1}=C_{L1}^{*}+\frac{C_{0,0}}{r}+\frac{C_{1,0}}{r^{2}}\;\text{cos}\;\theta , \end{array}$$ (45)

$$\begin{array}{c} R_{1}=g_{0}\left(t\right)+g_{1}\left(t\right)\;\text{cos}\;\theta , \end{array}$$ (46)

the growth velocity of the particle is written as

$$\begin{array}{c} U_{I}=\frac{dR_{0}}{dt}+\varepsilon \frac{dg_{0}}{dt}+\varepsilon \frac{dg_{1}}{dt}P_{1}^{0}\left(\text{cos}\;\theta \right), \end{array}$$ (47)

where

$$\begin{array}{ccc} & T_{S1}^{*}=\frac{\lambda_{t}r^{2}}{6}\frac{d(\frac{dR_{0}}{dt}R_{0})}{dt}, & \end{array}$$

$$\begin{array}{ccc} T_{L1}^{*}= & A_{m}\left(\frac{1}{8r^{3}}R_{0}^{3}+\frac{3}{4r}R_{0}-\frac{1}{2}\right)\frac{dR_{0}}{dt}R_{0}^{2}\;\text{cos}\;\omega t\;\text{cos}\;\theta \left(e^{Q_{0}-\rho }\right)+\frac{r}{2}\frac{d(\frac{dR_{0}}{dt}R_{0}^{2})}{dt}\left(e^{Q_{0}-\rho }\right)\\ & +\frac{r}{2}\left(\frac{dR_{0}}{dt}R_{0}^{2}\right)\frac{dQ_{0}}{dt}\left(e^{Q_{0}-\rho }\right)+C_{1}, & \end{array}$$

$$\begin{array}{cc} C_{L1}^{*}= & A_{m}\;\text{cos}\;\omega t\frac{dR_{0}}{dt}R_{0}^{2}\left(e^{Q_{0}-\rho }\right)\left(\lambda_{c}\left(S_{k}-S_{T}^{\left(1\right)}-S_{T}^{\left(3\right)}\right)-\left(S_{T}^{\left(1\right)}+S_{T}^{\left(3\right)}\right)\right)\left(\frac{1}{8r^{3}}R_{0}^{3}+\frac{3}{4r}R_{0}-\frac{1}{2}\right)\\ & \cdot \text{cos}\;\theta +\frac{r}{2}\left[\frac{d(\frac{dR_{0}}{dt}R_{0}^{2})}{dt}+\frac{dR_{0}}{dt}R_{0}^{2}\frac{dQ_{0}}{dt}\right]\left(e^{Q_{0}-\rho }\right)\left(\lambda_{c}\left(S_{k}-S_{T}^{\left(1\right)}-S_{T}^{\left(3\right)}\right)-\left(S_{T}^{\left(1\right)}+S_{T}^{\left(3\right)}\right)\right)\\ & -\frac{1}{2r^{2}}S_{T}^{\left(2\right)}\left(S_{k}-S_{T}^{\left(1\right)}-S_{T}^{\left(3\right)}\right){\left(\frac{dR_{0}}{dt}R_{0}^{2}\right)}^{2}{\left(e^{Q_{0}-\rho }\right)}^{2}+C_{2}. \end{array}$$

It shoud be pointed out that the first order approximation of the solutions obtained in Eqs (43) and (45) don’t satisfy the far-field condition Eq (41). This obvious contradiction is due to the influence of oscillatory flow. The viscous force is dominant in the area near the particles, and the inertial force is negligible, while the inertial force is dominant in the area far away from the particles, and the viscous force can be ignored. Because the leading order approximations Eqs (29)–(31) are independent of the inertia term (***U*** ⋅ ∇)***U***, it is not uniformly valid in the entire region. In order to solve this problem, we divide the entire melt region into an internal region close to the particles and an external region away from the particles. The asymptotic solution in Eq (13) is regarded as the interior solution of the interior domain. In the outer region, we consider the influence of inertial force. We find the external solutions of the temperature field and concentration field of the liquid phase $T_{L}^{\left(0\right)}$ and $C_{L}^{\left(0\right)}$ by using the asymptotic matching method.

$$\begin{array}{c} T_{L}^{\left(0\right)}=-\varepsilon +\varepsilon^{2}e^{-\frac{A_{m}\left|\text{cos}\;\omega t\right|}{2}\rho \;\text{cos}\;\theta }\sqrt{\frac{\pi }{A_{m}\rho }}\;\sum_{n=0}^{\infty }B_{n}\left(t\right)K_{n+\frac{1}{2}}(\frac{A_{m}}{2}\left|\text{cos}\;\omega t\right|\rho )P_{n}\left(\text{cos}\;\theta \right), \end{array}$$

$$\begin{array}{c} C_{L}^{\left(0\right)}=-\varepsilon +\varepsilon^{2}e^{-\frac{\lambda_{c}A_{m}\left|\text{cos}\;\omega t\right|}{2}\rho \;\text{cos}\;\theta }\sqrt{\frac{\pi }{\lambda_{c}A_{m}\rho }}\;\sum_{n=0}^{\infty }B_{n}\left(t\right)K_{n+\frac{1}{2}}(\frac{A_{m}\lambda_{c}}{2}\left|\text{cos}\;\omega t\right|\rho )P_{n}\left(\text{cos}\;\theta \right), \end{array}$$ (48)

We match the internal and external solutions of the temperature field and the concentration field to determine *C*_1_ and *C*_2_,

$$\begin{array}{c} C_{1}=\frac{1}{2}A_{m}\frac{dR_{0}}{dt}R_{0}^{2}\left|\text{cos}\;\omega t\right|, \end{array}$$

$$\begin{array}{c} C_{2}=-\frac{1}{2}A_{m}\frac{dR_{0}}{dt}R_{0}^{2}\left(\lambda_{c}\left(S_{K}-S_{T}^{\left(1\right)}-S_{T}^{\left(3\right)}\right)-\left(S_{T}^{\left(1\right)}+S_{T}^{\left(3\right)}\right)\right)\left|\text{cos}\;\omega t\right|, \end{array}$$

$$\begin{array}{cc} A_{0,0}= & R_{0}B_{0,0}+R_{0}\frac{dR_{0}}{dt}g_{0}\left(t\right)+R_{0}^{2}\frac{dR_{0}}{dt}\bar{g_{0}}\left(t\right)+\frac{\lambda_{t}R_{0}^{3}}{6}\frac{d}{dt}\left(R_{0}\frac{dR_{0}}{dt}\right)-\frac{R_{0}^{2}}{2}\frac{d}{dt}\left(R_{0}^{2}\frac{dR_{0}}{dt}\right)\\ & -\frac{R_{0}^{2}}{2}\frac{d}{dt}\left(R_{0}^{2}\frac{dR_{0}}{dt}\right)\frac{dQ_{0}}{dt}-C_{1}R_{0}, \end{array}$$

$$\begin{array}{c} A_{1,0}=R_{0}^{3}B_{1,0}+R_{0}^{2}\frac{dR_{0}}{dt}g_{1}\left(t\right)+R_{0}^{3}\frac{dR_{0}}{dt}\bar{g_{1}}\left(t\right)-\frac{3}{8}A_{m}\frac{dR_{0}}{dt}R_{0}^{4}\;\text{cos}\;\omega t, \end{array}$$

$$\begin{array}{cc} B_{0,0}= & \frac{2\Gamma }{R_{0}^{2}}g_{0}\left(t\right)-\frac{\lambda_{t}R_{0}^{2}}{6}\frac{d}{dt}\left(\frac{dR_{0}}{dt}R_{0}\right)-M_{c}C_{2}-M_{c}\frac{C_{0,0}}{R_{0}}-E^{-1}M_{K}\frac{dg_{0}\left(t\right)}{dt}-\frac{R_{0}M_{c}}{2}(\frac{d(\frac{dR_{0}}{dt}R_{0}^{2})}{dt}\\ & +\frac{dR_{0}}{dt}R_{0}^{2}\frac{dQ_{0}}{dt})\left(\lambda_{c}\left(S_{K}-S_{T}^{\left(1\right)}-S_{T}^{\left(3\right)}\right)-\left(S_{T}^{\left(1\right)}+S_{T}^{\left(3\right)}\right)\right)+\left(S_{K}-S_{T}^{\left(1\right)}-S_{T}^{\left(3\right)}\right)M_{c}\frac{dR_{0}}{dt}\\ & \cdot g_{0}\left(t\right)+\left(S_{K}-S_{T}^{\left(1\right)}-S_{T}^{\left(3\right)}\right)M_{c}\frac{dR_{0}}{dt}R_{0}\bar{g_{0}}\left(t\right)+\frac{M_{c}}{2R_{0}^{2}}S_{T}^{\left(2\right)}\left(S_{K}-S_{T}^{\left(1\right)}-S_{T}^{\left(3\right)}\right){\left(\frac{dR_{0}}{dt}R_{0}^{2}\right)}^{2}, \end{array}$$

$$\begin{array}{cc} B_{1,0}= & -\frac{3}{8}M_{c}A_{m}\;\text{cos}\;\omega t\frac{dR_{0}}{dt}R_{0}\left(\lambda_{c}\left(S_{K}-S_{T}^{\left(1\right)}-S_{T}^{\left(3\right)}\right)-\left(S_{T}^{\left(1\right)}+S_{T}^{\left(3\right)}\right)\right)-M_{c}\frac{C_{1,0}}{R_{0}^{3}}-E^{-1}M_{K}\\ & \cdot \frac{1}{R_{0}}\frac{dg_{1}\left(t\right)}{dt}+\left(S_{K}-S_{T}^{\left(1\right)}-S_{T}^{\left(3\right)}\right)\frac{M_{c}}{R_{0}}\frac{dR_{0}}{dt}g_{1}\left(t\right)+\left(S_{K}-S_{T}^{\left(1\right)}-S_{T}^{\left(3\right)}\right)\frac{dR_{0}}{dt}M_{c}\bar{g_{1}}\left(t\right), \end{array}$$

$$\begin{array}{cc} C_{0,0}= & S_{K}R_{0}^{2}\frac{dg_{0}\left(t\right)}{dt}+\frac{R_{0}^{2}}{2}\left(\frac{d(\frac{dR_{0}}{dt}R_{0}^{2})}{dt}+R_{0}^{2}\frac{dR_{0}}{dt}\frac{dQ_{0}}{dt}\right)\left(\lambda_{c}\left(S_{K}-S_{T}^{\left(1\right)}-S_{T}^{\left(3\right)}\right)\right)\\ & -S_{K}\frac{dR_{0}}{dt}R_{0}^{3}+2S_{K}g_{0}\left(t\right)\frac{dR_{0}}{dt}R_{0}+\left(S_{K}-S_{T}^{\left(1\right)}-S_{T}^{\left(3\right)}\right)R_{0}^{2}\frac{dR_{0}}{dt}\bar{g_{0}}\left(t\right)\\ & -\left(S_{T}^{\left(1\right)}+S_{T}^{\left(3\right)}\right)A_{0,0}+\left(S_{T}^{\left(1\right)}+S_{T}^{\left(3\right)}\right)R_{0}^{2}\frac{dR_{0}}{dt}\bar{g_{0}}\left(t\right)+S_{T}^{\left(2\right)}C_{L,\infty }R_{0}^{2}\frac{dR_{0}}{dt}, \end{array}$$

$$\begin{array}{cc} C_{1,0}= & \frac{R_{0}^{3}}{2}S_{K}\frac{dg_{1}\left(t\right)}{dt}-\frac{9}{16}A_{m}\;\text{cos}\;\omega t\frac{dR_{0}}{dt}R_{0}^{4}\left(\lambda_{c}\left(S_{K}-S_{T}^{\left(1\right)}-S_{T}^{\left(3\right)}\right)\right)+S_{K}\frac{dR_{0}}{dt}R_{0}^{2}g_{1}\left(t\right)\\ & +\left(S_{K}-S_{T}^{\left(1\right)}-S_{T}^{\left(3\right)}\right)\frac{R_{0}^{3}}{2}\frac{dR_{0}}{dt}\bar{g_{1}}\left(t\right)-\left(S_{T}^{\left(1\right)}+S_{T}^{\left(3\right)}\right)A_{1,0}+\left(S_{T}^{\left(1\right)}+S_{T}^{\left(3\right)}\right)\frac{R_{0}^{3}}{2}\frac{dR_{0}}{dt}\bar{g_{1}}\left(t\right), \end{array}$$

$$\begin{array}{cc} \frac{dg_{0}\left(t\right)}{dt}= & \frac{1}{N}\left(\frac{dR_{0}}{dt}R_{0}^{2}\right)(\frac{S_{K}A_{m}M_{c}\;\text{cos}\;\omega t\left(\lambda_{c}\left(S_{K}-S_{T}^{\left(1\right)}-S_{T}^{\left(3\right)}\right)-\left(S_{T}^{\left(\text{1}\right)}+S_{T}^{\left(3\right)}\right)\right)}{2}\\ & +M_{c}S_{K}^{2}+\frac{S_{K}A_{m}\left|\text{cos}\;\omega t\right|}{2})-\frac{1}{N}M_{c}S_{T}^{\left(2\right)}S_{K}C_{L,\infty }\left(\frac{dR_{0}}{dt}R_{0}\right)-\frac{1}{N}(M_{c}S_{K}^{2}g_{0}\left(t\right)\\ & +\left(1-M_{c}\left(S_{T}^{\left(1\right)}+S_{T}^{\left(3\right)}\right)\right)S_{K}g_{0}\left(t\right))\left(\frac{dR_{0}}{dt}\right)+\frac{1}{N}\frac{2\Gamma S_{K}}{R_{0}^{2}}g_{0}\left(t\right)+\frac{1}{N}\frac{\lambda_{t}R_{0}^{2}}{3}\\ & \cdot \left(1-M_{c}\left(S_{T}^{\left(1\right)}+S_{T}^{\left(3\right)}\right)\right)S_{K}k_{t}\frac{d(\frac{dR_{0}}{dt}R_{0})}{dt}+\frac{1}{N}\frac{M_{c}S_{K}R_{0}^{2}}{2}S_{T}^{\left(2\right)}\left(S_{K}-S_{T}^{\left(1\right)}-S_{T}^{\left(3\right)}\right)\\ & \cdot {\left(\frac{dR_{0}}{dt}\right)}^{2}-\frac{R_{0}}{N}(M_{C}S_{K}\left(\lambda_{C}\left(S_{K}-S_{T}^{\left(1\right)}-S_{T}^{\left(3\right)}\right)\right)+\frac{S_{K}\left(1-M_{C}\left(S_{T}^{\left(1\right)}+S_{T}^{\left(3\right)}\right)\right)}{2}\\ & +\frac{\left(S_{T}^{\left(1\right)}+S_{T}^{\left(3\right)}\right)\left(1-M_{C}\left(S_{T}^{\left(1\right)}+S_{T}^{\left(3\right)}\right)\right)}{2})\left(\frac{d(\frac{dR_{0}}{dt}R_{0}^{2})}{dt}+\frac{dR_{0}}{dt}R_{0}^{2}\frac{dQ_{0}}{dt}\right), \end{array}$$

where

$$\begin{array}{c} N=S_{K}\left(1-M_{c}\left(S_{T}^{\left(1\right)}+S_{T}^{\left(3\right)}\right)\right)R_{0}+M_{c}S_{K}^{2}R_{0}+S_{K}M_{K}E^{-1}, \end{array}$$

$$\begin{array}{cc} \frac{dg_{1}\left(t\right)}{dt}= & \frac{1}{G}[A_{m}S_{K}\;\text{cos}\;\omega t\left(\lambda_{c}\left(S_{K}-S_{T}^{\left(1\right)}-S_{T}^{\left(3\right)}\right)\right)\left(\frac{3}{16}k_{t}+\frac{3}{8}M_{c}\right)\\ & +\left(1-M_{c}\left(S_{T}^{\left(1\right)}+S_{T}^{\left(3\right)}\right)\right)A_{m}\;\text{cos}\;\omega t\left(\frac{9}{8}\left(S_{T}^{\left(1\right)}+S_{T}^{\left(3\right)}\right)-\frac{3}{4}S_{K}\right)]\left(\frac{dR_{0}}{dt}R_{0}^{2}\right)\\ & +\frac{1}{2G}\left[S_{K}^{2}M_{c}\left(k_{t}+2\right)\bar{g_{1}}\left(t\right)+2S_{K}\left(1-M_{c}\left(S_{T}^{\left(1\right)}+S_{T}^{\left(3\right)}\right)\right)\bar{g_{1}}\left(t\right)\right]\left(\frac{dR_{0}}{dt}R_{0}\right), \end{array}$$

where

$$\begin{array}{c} G=\frac{\left[2S_{K}\left(1-M_{c}\left(S_{T}^{\left(1\right)}+S_{T}^{\left(3\right)}\right)\right)R_{0}+\left(k_{t}+2\right)\left(S_{K}^{2}M_{c}R_{0}+2S_{K}E^{-1}M_{K}\right)\right]}{2}. \end{array}$$

From the above calculations, we can obtain the uniformly valid asymptotic solutions for the temperature fields and the concentration field

$$\begin{array}{c} R=R_{0}+\varepsilon R_{1}+O\left(\varepsilon^{2}\right),\;\;C_{L}=\varepsilon \lambda_{c}C_{L0}+\varepsilon^{2}\lambda_{c}C_{L1}+O\left(\varepsilon^{3}\right),\\ T_{L}=\varepsilon T_{L0}+\varepsilon^{2}T_{L1}+O\left(\varepsilon^{3}\right),\;T_{S}=\varepsilon T_{S0}+\varepsilon^{2}T_{S1}+O\left(\varepsilon^{3}\right), \end{array}$$ (49)

and the growth velocity of the particle is

$$\begin{array}{c} U_{I}=\frac{dR_{0}}{dt}+\varepsilon \frac{dR_{1}}{dt}+O\left(\varepsilon^{2}\right). \end{array}$$ (50)

## Discussion

The mathematical model of spherical crystal growth in binary alloy melt has been studied by using the multi-scale method. We obtained approximate analytical solutions of liquid phase temperature, solid phase temperature, solute concentration and interfacial growth rate of the spherical crystal growth model. In actual operation, the oscillatory flow in the melt is caused by the vibration acceleration *a* = −Ω*A*_*D*_ and the angular frequency Ω = 2*πf*, so the vibration acceleration and the angular frequency are selected as the control parameters of the oscillatory flow. In the following, the effect of Soret effect on the growth of spherical crystals in binary alloy melts is analyzed by using the physical parameters in Table 1 and the asymptotic solutions Eqs (43)–(46). In order to analyze the effect of Soret coefficient *S*_*T*_ = *D*_*T*_/*D*_*L*_ on the crystal growth rate *U*_*I*_, the *dR*_0_/*dt* in Eq (32) is solved as

$$\begin{array}{c} \frac{dR_{0}}{dt}=\frac{R_{0}-2\Gamma }{R_{0}^{2}+R_{0}E^{-1}M_{k}+R_{0}^{2}M_{c}S_{k}+R_{0}^{2}\frac{D_{L}}{\kappa_{L}}\left(\beta_{0}m+\beta_{1}\Delta T\right)S_{T}}. \end{array}$$ (51)

**Fig 1:**
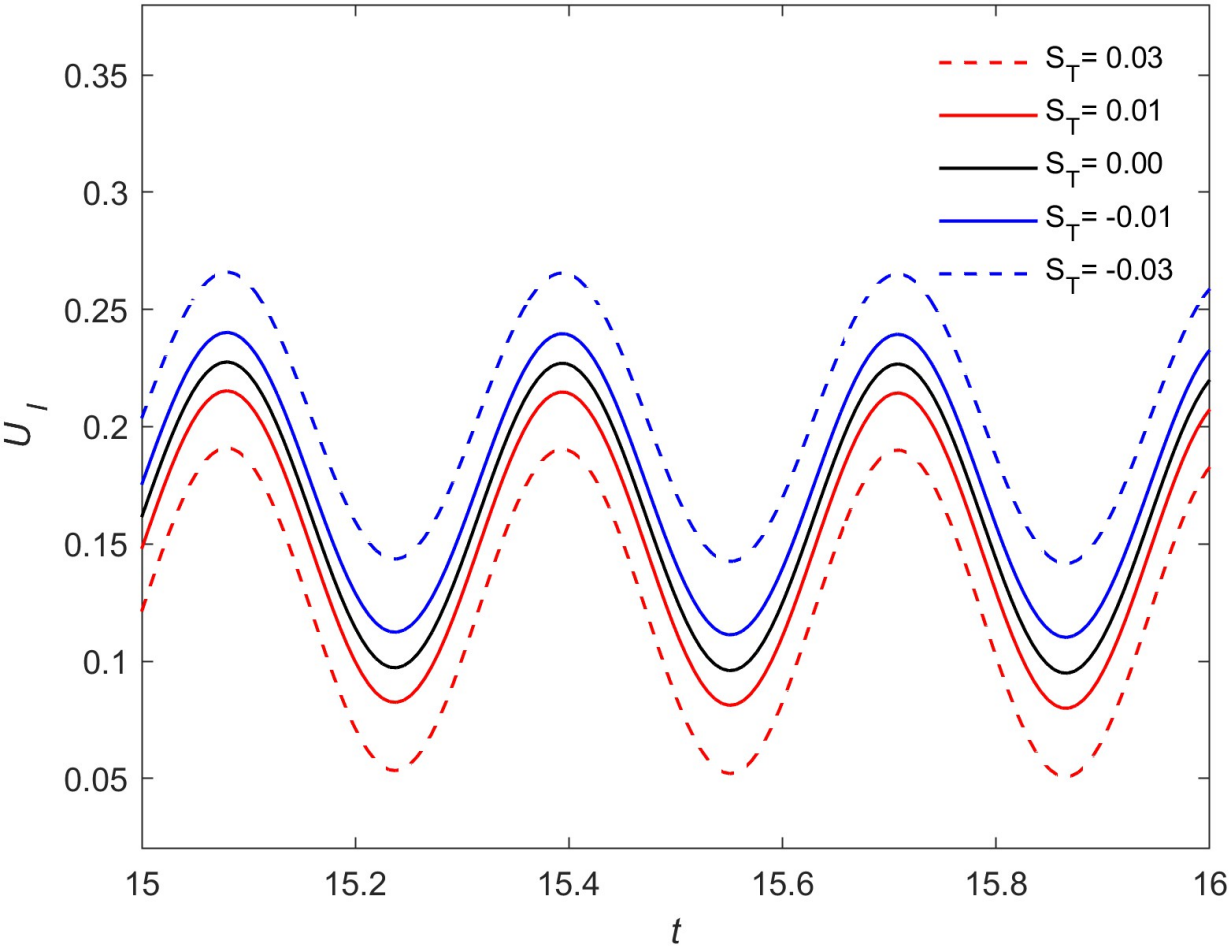
Dependence of the growth velocity of the particle on the Soret coefficients. The interface velocity curves at a fixed frequency *f* = 50Hz, acceleration *a* = 9000 m/s^2^ and undercooling Δ*T* = 19K for the different values of Soret coefficients *S*_*T*_ = -0.03, -0.01, 0, 0.01, 0.03 (from up to down), where *ε*=0.053, λ_*t*_=5.178, *E*=0.0105, *M*_*K*_=0.01613, Γ=0.4754, λ_*c*_=27.303.

It is evident that the positive and negative values of (*β*_0_*m* + *β*_1_Δ*T*)*S*_*T*_ affect the growth rate of the crystal interface. The effect of the Soret coefficient on the crystal growth rate *U*_*I*_ in the Cu-Fe dilute alloy melt (*β*_0_ = 0, *β*_1_ = 1) of Δ*T* = 19 K is shown below. Without loss of generality, when *f* = 50Hz, *a* = 9000 m/s^2^, Fig 1 shows the relationship between crystal growth rate and time from *t* = 15 to *t* = 16. It can be seen that the crystal growth rate oscillates with the flow. When the Soret coefficient *S*_*T*_ is positive, the crystal growth rate decreases with the increase of *S*_*T*_. When the Soret coefficient *S*_*T*_ is negative, the growth rate of the crystal increases with the decrease of *S*_*T*_. This conclusion is consistent with the relationship between *U*_*I*_ and *S*_*T*_ in Eq (51). Physically, the growth rate of an interface is primarily governed by the heat and solute fluxes near it. The Soret effect, induced by temperature gradients, alters the value of flow velocity near the interface [25], resulting in changes to the solute flux entering the interface. This suggests that the Soret effect influences the balance of heat and solute fluxes near the particle interface, thereby modifying the particle growth rate.

**Table 1:** Thermophysical properties of copper-iron alloys [31, 32].

Parameter	Cu-Fe	Unit
**Initial concentration** *C*_∞_	3	wt%
**Melting temperature** *T*_*M*_	1810	K
**Liquid thermal conductivity** *k*_*L*_	160	J m^−1^s^−1^K^−1^
**Solid thermal conductivity** *k*_*S*_	30.9	J m^−1^s^−1^K^−1^
**Isothermal diffusion coefficient** *D*_*L*_	1.1 × 10^−7^	m^2^ s^−1^
**Latent heat** Δ*H*	2.4 × 10^9^	J m^−3^
**Density** *ρ*	7940	kg m^−3^
**Heat capacity** *c*_*p*_	843	J kg^−1^K^−1^
**Liquidus slope** *m*	3.26	K wt%
**Segregation coefficient** *k*	1.4	−

Parameter represents the name of each physical parameter, the second column represents the value of the physical parameter, and the third column represents the unit corresponding to the physical parameter.

**Fig 2:**
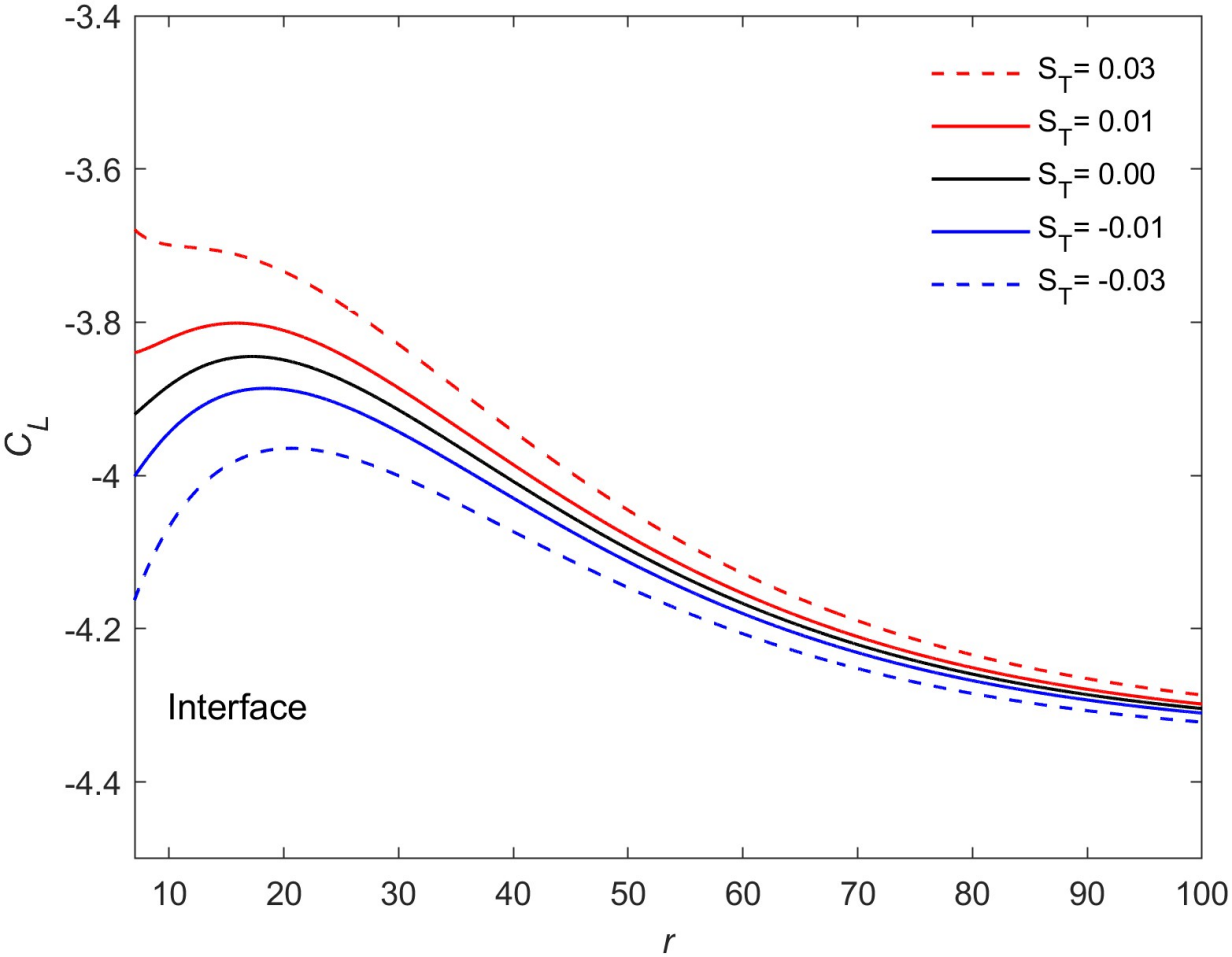
Dependence of solute concentration on the Soret coefficients. The interface solute concentration at a fixed frequency *f* = 50Hz, acceleration *a* = 9000 m/s^2^, *t* = 11.29s and undercooling Δ*T* = 19K for the different values of Soret coefficients *S*_*T*_ = 0.03, 0.01, 0, -0.01, -0.03 (from up to down), where *ε*=0.053, *E*=0.0105, *M*_*K*_=0.01613, Γ=0.4754, λ_*c*_=27.303, λ_*t*_=5.178.

**Fig 3:**
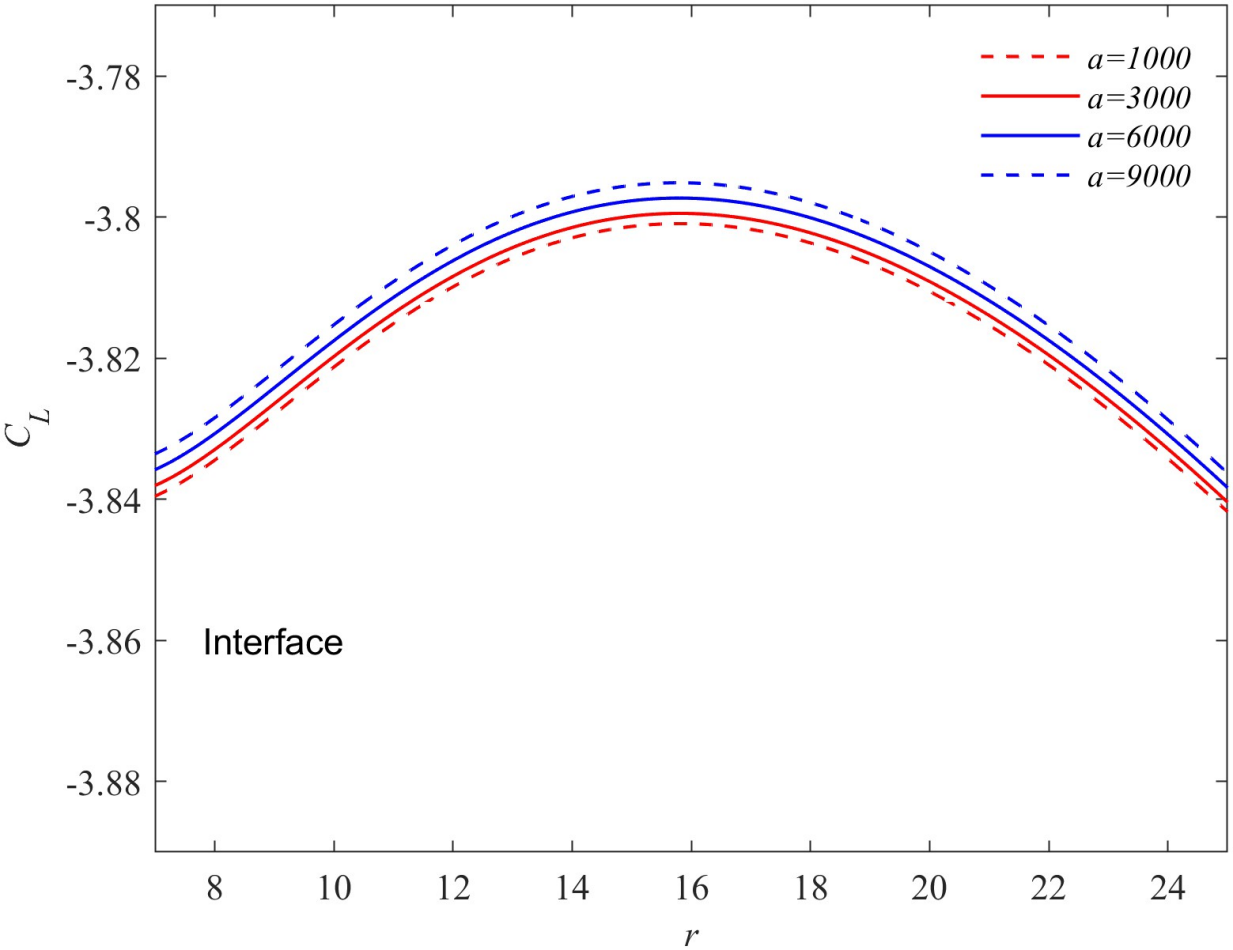
Concentration profiles of a particle in the liquid phase at different values of acceleration, where *S*_*T*_=0.01. The interface solute concentration at a fixed frequency *f* = 50Hz, *t* = 11.29s and undercooling Δ*T* = 19K for the different values of acceleration *a* = 9000 m/s^2^, *a* = 6000 m/s^2^, *a* = 3000 m/s^2^, *a* = 1000 m/s^2^ (from up to down), where *ε*=0.053, *E*=0.0105, *M*_*K*_=0.01613, Γ=0.4754, λ_*c*_=27.303, λ_*t*_=5.178.

**Fig 4:**
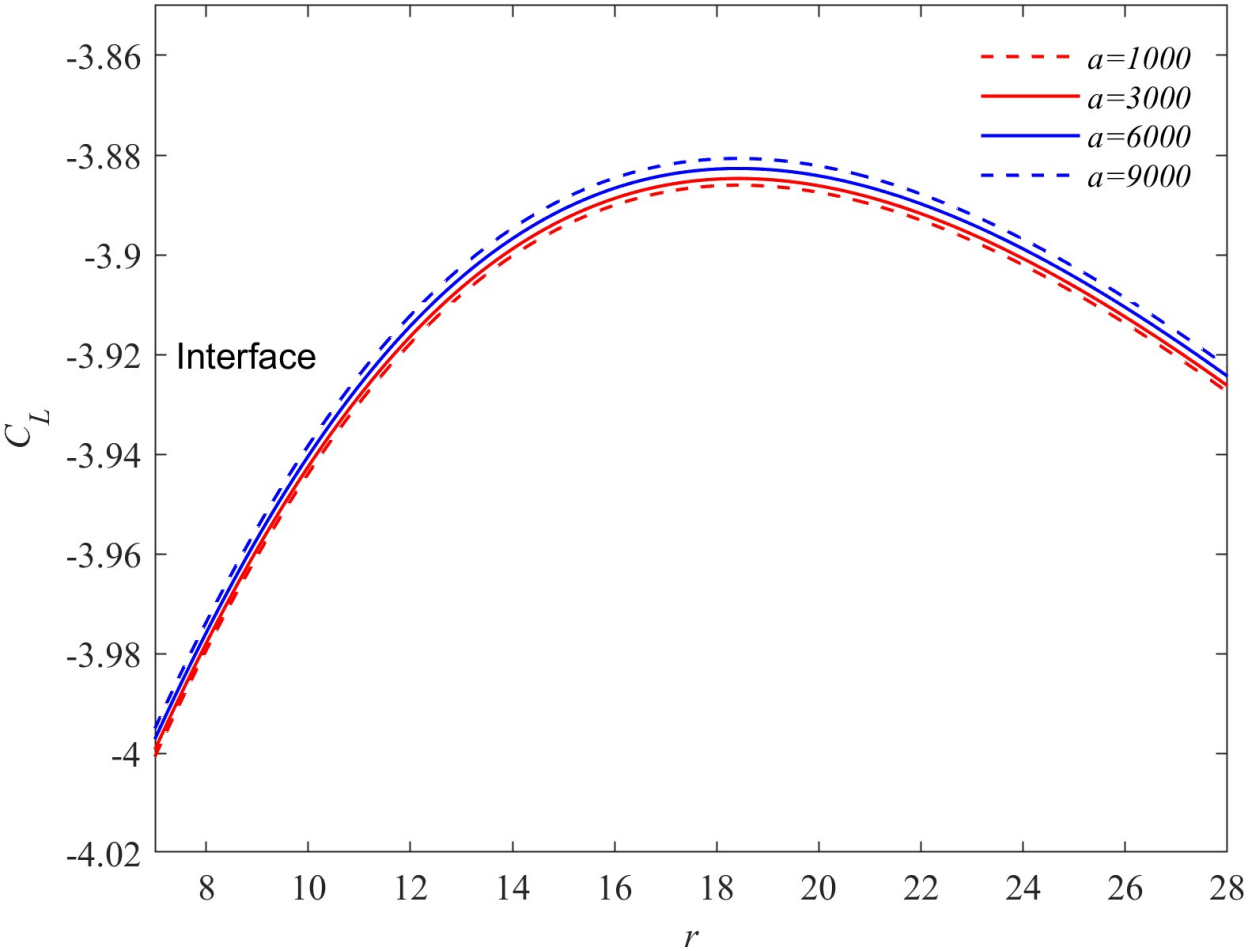
Concentration profiles of a particle in the liquid phase at different values of acceleration, where *S*_*T*_=-0.01. The interface solute concentration at a fixed frequency *f* = 50Hz, *t* = 11.29s and undercooling Δ*T* = 19K for the different values of acceleration *a* = 9000 m/s^2^, *a* = 6000 m/s^2^, *a* = 3000 m/s^2^, *a* = 1000 m/s^2^ (from up to down), where *ε*=0.053, *E*=0.0105, *M*_*K*_=0.01613, Γ=0.4754, λ_*c*_=27.303, λ_*t*_=5.178.

The variation of solute concentration of the crystal with the Soret coefficient under action of oscillatory flow (*f* = 50Hz, *a* = 9000 m/s^2^) is presented in Fig 2 when Δ*T* = 19K, *t* = 11.29. As shown in the figure that ∂*C*_*L*_/∂*r* > 0 is near the crystal interface and ∂*C*_*L*_/∂*r* < 0 is far away from the interface. This means that when the solute flux flows from the liquid phase into the interface, it fluctuates due to the influence of the oscillatory flow. Consequently, the solute concentration near the interface is higher than that in the far field, and the solute concentration reaches its maximum value not far from the interface. When the Soret coefficient is positive, the solute flux entering the interface decreases with the increase of Soret coefficient, so that the solute concentration near the interface increases with the increase of Soret coefficient. When the Soret coefficient is negative, the solute flux entering the interface increases with the decrease of Soret coefficient, so that the solute concentration near the interface decreases with the decrease of Soret coefficient. Physically, the Soret effect can be considered as a nonequilibrium cross-flow effect between mass and heat transport in fluid mixtures [33]. Local thermodynamic equilibrium provides the best framework for describing this effect [34]. Consequently, Fig 2 can be understood as illustrating how the Soret effect controls thermodynamic disequilibrium near the interface, influencing the solute flux entering the interface from the liquid phase and and determining the uniformity of particle composition. Figs 3 and 4 illustrate the effects of different oscillating accelerations on the solute concentration near the interface (Δ*T* = 19K, *f* = 50Hz, *t* = 11.29). Based on Fig 2, it can be concluded that oscillatory flow enhances the Soret effect. Researches by Zhang et al. [35] and Brover et al. [36] has demonstrated that the Soret effect facilitates grain refinement. Additionally, Chen et al. [29] have shown that oscillatory flow alternately promotes and inhibits grain growth. Increasing the amplitude of oscillation leads to further grain refinement. From the above analysis, it is evident that the Soret effect has a significant impact on the kinetic processes near the crystal interface. Moreover, the combined action of the Soret effect and oscillatory flow can substantially accelerate the refinement of grains.

## Conclusion

This study investigates the influence of the Soret effect on the growth of spherical crystals in undercooled alloy melts subjected to oscillatory flow. We have developed a comprehensive mathematical and physical model for spherical crystal growth that incorporates the Soret effect. Using the multiple scale method, we derive approximate analytical expressions for the temperature field, concentration field, interface radius and crystal growth rate. The model and corresponding solutions provide a detailed framework for understanding how the Soret effect impacts crystal growth dynamics under oscillatory flow. Our analysis reveals significant insights into the relationship between the Soret effect and crystal growth behaviors.

1. **Impact of the Soret Effect:** Our results reveal that the Soret effect significantly affects the crystal growth rate and solute concentration at the interface. Specifically,
  - **Positive Soret Coefficient:** Leads to a decrease in the crystal growth rate as the Soret coefficient increases, while the solute concentration near the interface increases.
  - **Negative Soret Coefficient:** Results in an increase in the crystal growth rate as the Soret coefficient decreases, and a decrease in the solute concentration near the interface.
2. **Role of Oscillatory Flow:** We demonstrated that low-frequency oscillatory flow amplifies the Soret effect. This amplification enhances the grain refinement effect associated with the Soret effect, providing a new perspective on controlling crystal structure through flow conditions.

These findings contribute to a deeper understanding of how thermal diffusion and oscillatory flow interact to influence crystal growth in alloy melts, offering potential avenues for optimizing material properties in practical applications.

## Supporting information

S1 Appendix Nomenclature. The meanings of symbols are presented in the Nomenclature.
(PDF)

S1 Dataset Minimal data set. (XLSX)
