# Supplementary material for: Effect of Soret diffusion on the growth of spherical crystals in supercooled alloy melts under oscillatory flow
Source: PLoS One. 2024 Nov 4;19(11):e0313150. doi: 10.1371/journal.pone.0313150 (PMC11534215; doi:10.1371/journal.pone.0313150)
Supplement: S1 Appendix — The meanings of symbols are presented in the Nomenclature. (PDF) [file pone.0313150.s001.pdf]

Nomenclature

ΔH	latent heat per unit volume
ΔT	undercooling
Γ	surface tension parameter
μ	interface kinetic coefficient
Pr	Prandtl number
υ	kinematical viscosity
ε	dimensionless relative undercooling parameter
$\boldsymbol{n}$	unit normal vector to the interface
$\boldsymbol{U}_L$	flow field velocity
C_∞	far-field concentration of the liquid phase
C_L	concentration of the liquid phase
C_S	concentration of the solid phase
k	solute segregation coefficient
m	liquidus slope in phase diagram
T_∞	far-field temperature of the liquid phase
T_L	temperature of the liquid phase
T_S	temperature of the solid phase
κ_S	thermal diffusivity in the solid phase
κ_L	thermal diffusivity in the liquid phase
λ_c	ratio of thermal diffusivity to solute diffusivity in the liquid phase
ρ_L	melt density
C_e	liquid phase equilibrium concentration of the flat interface
c_p	specific heat
k_t	ratio of thermal parameters
M_C	morphological number
M_K	interfacial kinetics parameter
T_e	liquid phase equilibrium temperature of the flat interface
T_I	temperature at the interface
U_I	local velocity of the crystal-melt interface
